# Janus MoSH/WSi_2_N_4_ van der Waals Heterostructure: Two-Dimensional Metal/Semiconductor Contact

**DOI:** 10.3390/molecules29153554

**Published:** 2024-07-28

**Authors:** Yongdan Wang, Xiangjiu Zhu, Hengshuo Zhang, Shitong He, Ying Liu, Wenshi Zhao, Huilian Liu, Xin Qu

**Affiliations:** 1 Key Laboratory of Functional Materials Physics and Chemistry of the Ministry of Education, Jilin Normal University, Changchun 130103, China; ydwang@jlnu.edu.cn (Y.W.); zxj1474131433@126.com (X.Z.); jlnuzhs@163.com (H.Z.); m15333854769@163.com (S.H.); 233120967@mails.jlnu.edu.cn (Y.L.); 2405006@jlnu.edu.cn (W.Z.); 2 School of Foreign Languages, Jilin Normal University, Siping 136000, China; 3 State Key Laboratory of Superhard Materials, College of Physics, Jilin University, Changchun 130012, China

**Keywords:** two-dimensional heterostructures, first-principles calculations, electronic properties, electrical contact

## Abstract

Constructing heterostructures from already synthesized two-dimensional materials is of significant importance. We performed a first-principles study to investigate the electronic properties and interfacial characteristics of Janus MoSH/WSi_2_N_4_ van der Waals heterostructure (vdWH) contacts. We demonstrate that the p-type Schottky formed by MoSH/WSi_2_N_4_ and MoHS/WSi_2_N_4_ has extremely low Schottky barrier heights (SBHs). Due to its excellent charge injection efficiency, Janus MoSH may be regarded as an effective metal contact for WSi_2_N_4_ semiconductors. Furthermore, the interfacial characteristics and electronic structure of Janus MoSH/WSi_2_N_4_ vdWHs can not only reduce/eliminate SBH, but also forms the transition from p-ShC to n-ShC type and from Schottky contact (ShC) to Ohmic contact (OhC) through the layer spacing and electric field. Our results can offer a fresh method for optoelectronic applications based on metal/semiconductor Janus MoSH/WSi_2_N_4_ vdW heterostructures, which have strong potential in optoelectronic applications.

## 1. Introduction

Innovations and continuous advancements in materials technology have enabled the successful synthesis of graphene, marking a significant milestone in the development of two-dimensional (2D) materials [1]. Graphene [2,3,4,5,6,7] was the first material to advance research in the two-dimensional field, and it possesses many remarkable qualities. However, the lack of a band gap in graphene [3] prevents it from being used in high-speed electronic devices, like field-effect transistors [8]. As a result, a two-dimensional semiconductor material with extraordinary properties and application potential has been intensively sought by science. A few years ago, due to their distinctive characteristics, two-dimensional (2D) materials [9,10,11] became the most alluring materials. Numerous 2D materials, such as transition metal dichalcogenides (TMDs) [12,13], phosphorene [14], and transition metal monochalcogenides (TMMs) [15,16], have been predicted and successfully synthesized up to this point. These two-dimensional materials possess a multitude of remarkable features, which positions them as potentially advantageous candidates for use in energy storage [17], photocatalysis [18], and optoelectronic applications [19,20,21]. 

Forming electrical contacts between metals and semiconductors is an important component in today’s electronic and optoelectronic devices. The direct result of electrical contact is that the functionality of electronic devices is effectively improved, thereby increasing the electron transfer efficiency [22] of semiconductors, which is a mutually beneficial situation. The investigation of the interactions between metals and semiconductors at interfaces is an absolutely necessary step in the process of developing highly effective and powerful electronic devices [23,24]. If the metal and semiconductor contacts are poor, the device may be damaged and rendered inoperable. The potential barrier usually generated at the interface semiconductor and metal is called the Schottky barrier (SB). The existence of a substantial Schottky barrier height (SBH) [25,26] can be a significant hindrance to charge injection efficiency in optoelectronic and electronic applications. Therefore, reducing or removing the Schottky barrier height (SBH) from the Schottky contact to the Ohmic contact in the metal–semiconductor junction (MSJ) is crucial for the successful development of effective and powerful nanodevices. Since most two-dimensional metal-semiconductor interfaces seen in nature are Schottky interfaces, there are both inherent and extrinsic constraints [27,28]. These limits include work function mismatch, surface defects, sustainable doping techniques, and many others. Therefore, the transition from ShC to OhC is an endeavor that is indeed a challenging task.

Very recently, researchers have been very interested in 2D materials with Janus structures because of the broken mirror symmetry of these materials, which leads to many novel features, such as enhanced piezoelectric properties, increased catalytic activity, and improved electronic transport characteristics [29,30]. Transition metal dichalcogenides (TMDs) are a class of single-layer structured materials known for their diverse physical properties, such as high carrier mobility, strong light–matter interactions, and flexibility [31,32,33]. These properties make TMDs highly suitable for use in transistors, photodetectors, and flexible electronics, which are of particular interest. In recent experiments, many Janus structures have been successfully synthesized with different synthetic strategies [34,35]. Cheng et al. [36] were the first to present the Janus structure of TMDs in 2013. Janus MoSSe monolayers were effectively created by thermal selenization and chemical vapor deposition by Lu et al. [35]. Recently, Janus MoSH was produced by controlled H_2_-plasma treatment [37]. It is anticipated that Janus MoSH will be dynamically stable at room temperature. As a result of its metallic characteristics and high inherent carrier concentration, it offers a great deal of potential for use in applications involving metal contacts in 2D semiconductor nanodevices [37].

Very recently, 2D layered MoSi_2_N_4_ and WSi_2_N_4_ have been successfully prepared experimentally by chemical vapor deposition (CVD) [38]. Researchers explored their theoretical electrical and optical properties [39,40,41] and found that 2D layered MoSi_2_N_4_ and WSi_2_N_4_ have semiconducting properties, good air stability at room temperature, and excellent mechanical strength [42]. However, compared with MoSi_2_N_4_, WSi_2_N_4_ has a larger band gap and higher molar mass, and at the same time shows a wider and stronger visible light absorption range and intensity and higher electron–hole separation in water [43]. In addition, we reviewed a large amount of information and found that in the research work on the formation of heterostructures between WSi_2_N_4_ and metals, except for the related articles on the formation of heterostructures in contact with graphene [44], there is no related research on the formation of heterostructures between WSi_2_N_4_ and other metals [45]. Therefore, we predict that WSi_2_N_4_ should also have broad application prospects [38].

More intriguingly, van der Waals heterostructures that are vertically stacked are thought to be a useful method for regulating characteristics and extending the range of possible applications for 2D materials. The electrical and optical characteristics of heterostructures depend heavily on the stacking arrangement and interfaces. Depending on the degree of contact between the two-layer materials, the ability to generate different stacked lattice orientations gives the heterostructure interface controllable physical features [46,47]. In this work, we used first-principles calculations to construct and study the electronic properties of metallic Janus MoSH and semiconductor WSi_2_N_4_ van der Waals heterostructures (vdWHs) and studied their interfacial properties under the layer distance and external electric field. Because of their broken vertical symmetry, Janus MoSH and WSi_2_N_4_ form contacts that result in two distinct surfaces: MoSH/WSi_2_N_4_ and MoHS/WSi_2_N_4_, respectively. The heterostructure remains energetically viable through the weak van der Waals interaction between metallic Janus MoSH and semiconducting WSi_2_N_4_ monolayers, which also maintains the intrinsic properties of the two monolayer materials. The findings demonstrate that Janus MoSH/WSi_2_N_4_ has an adjustable SBH, and the contact type can be switched from p-type ShC to n-type ShC and from ShC to OhC. Janus MoSH/WSi_2_N_4_ vdWHs have potential applications in high-performance optoelectronic devices such as photodetectors, light-emitting diodes (LEDs), and solar cells. The ability to engineer the Schottky barrier height (SBH) and transition between Schottky and Ohmic contacts makes these heterostructures particularly attractive for improving charge injection efficiency and overall device performance. Our research can offer a fresh method for optoelectronic applications based on Janus MoSH/WSi_2_N_4_ vdWHs. This method of constructing metal–semiconductor heterostructures has broad application prospects in Schottky electronic devices and provides a foundation for the practical development of advanced optoelectronic devices.

## 2. Results and Discussion

### 2.1. Geometric Structures and Electronic Properties

**Figure 1:**
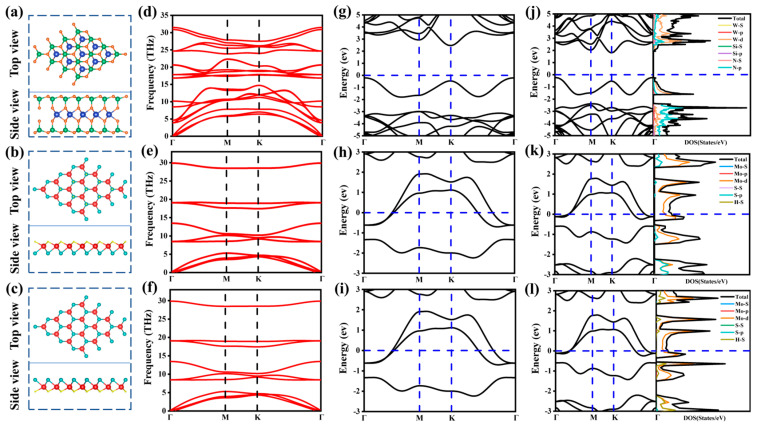
(**a**–**c**) Optimized monolayers WSi_2_N_4_ and Janus MoSH (MoHS) top and side panels. (**d**–**f**) Phonon dispersion curve. (**g**–**i**) HSE06 method projected band structures of the WSi_2_N_4_ and Janus MoSH (MoHS), respectively. (**j**–**l**) PBE method projected band structures and state density of the WSi_2_N_4_ and Janus MoSH (MoHS), respectively. The blue, green, orange, red, cyan, and yellow balls represent tungsten, silicon, nitrogen, molybdenum, sulfur, and hydrogen atoms, respectively.

Figure 1 shows the atomic structure, phonon spectrum, projected band structure, and density of states of the semiconducting WSi_2_N_4_ and Janus metallic MoSH (MoHS) monolayers, respectively. After geometry optimization, both WSi_2_N_4_ and Janus MoSH (MoHS) alone exhibit layered atomic structures with lattice constants of 2.91 Å, 3.18 Å, and 3.18 Å, which are consistent with the values [37,48] obtained by previous experimental measurements and theoretical calculations. As shown in Figure 1a–c, for the optimized WSi_2_N_4_ geometry structure, the W-N_2_ layer is sandwiched between the Si-N bilayer in the WSi_2_N_4_ monolayer, while in the Janus MoSH (MoHS) geometry structure, Mo atoms are sandwiched between H and S atoms. In addition, it can be observed from Figure 1h,i that the energy band passes through the Fermi level (“Fermi level” appears in the following articles and is represented by “E_F_”), and the monolayer Janus MoSH (MoHS) exhibits metallic properties, while in Figure 1g,j, HSE06 (Heyd–Scuseria–Ernzerhof) and PBE (Perdew, Burke, and Ernzerhof) methods are used to calculate the energy bands. It can be found that the WSi_2_N_4_ monolayer exhibits semiconductor characteristics. It is an indirect bandgap semiconductor. The Γ point and the K point are the locations of the valence band maximum (VBM) and conduction band minimum (CBM). The bandgap value of HSE06 is 2.66 eV, and the PBE is 2.03 eV. Generally speaking, HSE06 can forecast bandgap values more accurately than PBE methods, which frequently overestimate the bandgaps of 2D semiconductors [49,50]. However, compared with the HSE06 method, the PBE bandgap of the WSi_2_N_4_ monolayer is more consistent with the experimental value, and the CBM and VBM position cannot be altered using the HSE06 approach [51]. Therefore, we adopt the PBE method for all calculations below. Furthermore, the density of states of semiconductor WSi_2_N_4_ and metal Janus MoSH (MoHS) is shown in Figure 1j–l. For the metal Janus MoSH, it can be found that the d-orbital contribution of Mo is dominant, followed by the p-orbital contribution of S, while for Janus MoHS, the d-orbital contribution of Mo is dominant, followed by the s-orbital contribution of H. For the semiconductor WSi_2_N_4_, the p-orbital contribution of N dominates the VBM, while the d-orbital contribution of W dominates the CBM. Furthermore, Figure 1d–f illustrate the phonon dispersion curves for the three monolayers under consideration. The presence of positive frequencies at the Γ point in monolayer WSi_2_N_4_ and the absence of any negative frequencies in Janus MoSH (MoHS) confirm the dynamic stability of the system.

### 2.2. Structures and Electronic Properties of Heterostructures

**Figure 2:**
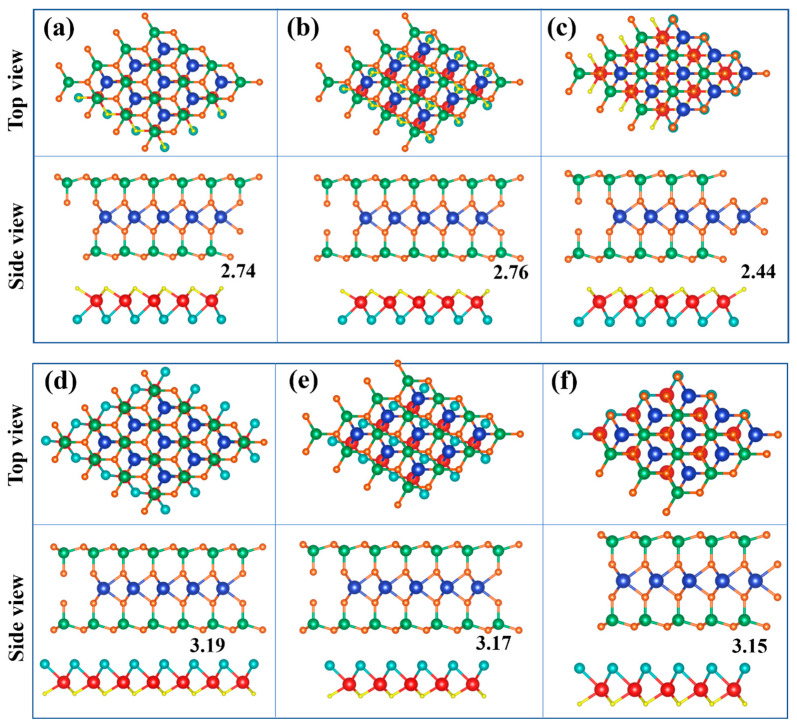
(**a**–**f**) Three different stacking forms of MoSH/WSi_2_N_4_, MoHS/WSi_2_N_4_ vdWHs, respectively. The blue, green, orange, red, cyan, and yellow balls represent tungsten, silicon, nitrogen, molybdenum, sulfur, and hydrogen atoms, respectively.

The heterostructure was formed by vertically stacking a single layer of WSi_2_N_4_ on top of a single layer of Janus MoSH (MoHS) along the z-direction. The initial equilibrium interlayer distances were set to 2.74 Å and 3.15 Å, respectively, which exceed the sum of the covalent radii of N atoms and H (S) atoms, indicating the absence of covalent bonding between the two monolayers comprising the system. At the same time, we take into account three different stacking configurations AA, AB, AC for the formation of two heterostructures MoSH/WSi_2_N_4_, MoHS/WSi_2_N_4_, corresponding to Figure 2a–f. As seen in Figure 2, in AA stacking, the two monolayers are completely corresponding, and W atoms are directly above the H (S) atoms; in AB-stacking, Mo atoms are between the W-Si atoms; in AC stacking, Mo atoms are in the hollow sites of monolayer WSi_2_N_4_. According to the calculation results, the energies of the three stacked configurations of MoSH/WSi_2_N_4_ and MoHS/WSi_2_N_4_ are very similar, and the energies in Figure 2a,f are relatively low, with Eb being −84.30725 eV and −84.32245 eV, respectively. Therefore, we construct heterostructures with (1 × 1) MoSH, (1 × 1) WSi_2_N_4_ and (1 × 1) MoHS, (1 × 1) WSi_2_N_4_ unit cells using AA, AC stacking methods. According to the formula m − n/m + n < 5% (m and n are the lattice constants of single-layer MoSH (MoHS) and WSi_2_N_4_, respectively), the lattice constants of MoSH/WSi_2_N_4_ and MoHS/WSi_2_N_4_ vdWHs are calculated to be 2.91 Å, and the lattice mismatch is 4.4% < 5%, which proves the rationality of the heterostructure. 

Additionally, we calculated the binding energy and confirmed the stability of the structure: E_b_ = E_vdWHs_ − E_MoSH(MoHS)_ − EWSi_2_N_4_, where E_vdWHs_, E_MoSH(MoHS)_ and EWSi_2_N_4_ are the sum energies of the corresponding vdWHs and MoSH(MoHS) and WSi_2_N_4_ monolayers, respectively. The binding energies of MoSH/WSi_2_N_4_ and MoHS/WSi_2_N_4_ are −0.43 eV and −0.18 eV, respectively. Since the binding energies of heterostructures have a negative sign, they are energetically stable. Furthermore, we also calculated the elastic constants of MoSH/WSi_2_N_4_ and MoHS/WSi_2_N_4_ vdWHs to assess the mechanical stability. The elastic constants C_11_, C_12_, and C_66_ = (C_11_ − C_12_)/2 of MoSH/WSi_2_N_4_ vdWHs are calculated to be 722 N/m, 203 N/m, and 259 N/m. At the same time, the elastic constants C_11_, C_12_, and C_66_ = (C_11_ − C_12_)/2 of MoHS/WSi_2_N_4_ are calculated to be 708 N/m, 221 N/m, and 243 N/m. The fact that the elastic constants of vdWHs, C_11_ > C_12_ and C_66_ > 0, satisfy the Born–Huang criterion [52,53] proves that vdWHs are stable. Furthermore, we also calculate the Young’s modulus and Poisson’s ratio of Y = (C_11_^2^ − C_12_^2^)/C_11_, V = C_12_/C_11_ and other systems. Figure S1 of the Supporting Information depicts a polar plot of Young’s modulus and Poisson’s ratio for vdWHs. The average Young’s modulus of MoSH/WSi_2_N_4_ vdWHs is 664 N/m and Poisson’s ratio is 0.28, while the average Young’s modulus of MoHS/WSi_2_N_4_ vdWHs is 639 N/m and Poisson’s ratio is 0.31, both of which are higher than those of graphene [51]. The results show that high in-plane stiffness is possessed by the two vdWHs.

**Figure 3:**
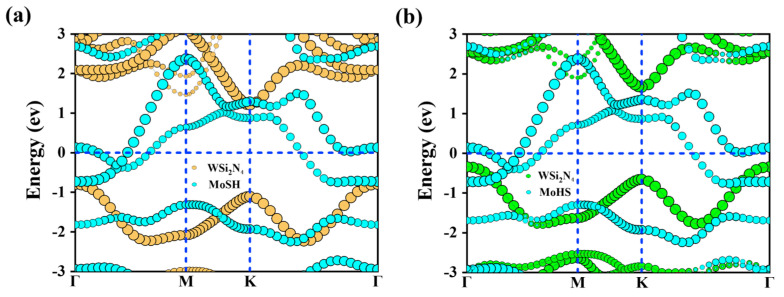
(**a**,**b**) Weighted projected band structures of MoSH/WSi_2_N_4_, MoHS/WSi_2_N_4_ vdWHs obtained by PBE calculations, respectively.

**Figure 4:**
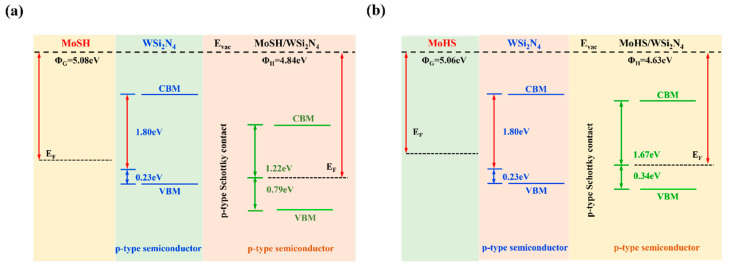
(**a**,**b**) The work functions of Janus MoSH (MoHS), WSi_2_N_4_ monolayer and their vdWHs.

The energy band structures [54] of MoSH/WSi_2_N_4_, MoHS/WSi_2_N_4_ vdWHs are shown in Figure 3a,b. MoSH (MoHS) and WSi_2_N_4_ maintain the original intrinsic band structure while forming heterostructures. Both the metallic and semiconducting properties of the Janus MoSH (MoHS) monolayer and WSi_2_N_4_ monolayer are well preserved. It is crucial to determine whether metal/semiconductor interactions form ShC or OhC [55,56,57]. The energy band structures in Figure 3a,b indicate that the Janus MoSH/WSi_2_N_4_ vdWHs form ShC, and we found that thebandgap value leading to PBE is 2.01 eV. The determination of the n-type or p-type ShC is widely recognized to be based on the SBH, as described by the Schottky–Mott rule [58]. Specifically, the SBH for the n-type ShC (Φ_Bn_) is determined by the difference between the CBM and the E_F_, denoted as Φ_Bn_ = E_CBM_ − E_F_. Similarly, the SBH for the p-type ShC (Φ_Bp_) is determined by the difference between the E_F_ and the VBM, denoted as Φ_Bp_ = E_F_ − E_VBM_. Furthermore, to verify the formation of ShC in these two heterostructures, we elucidated the work functions of monolayers of metallic Janus MoSH and semiconductor WSi_2_N_4_, along with their corresponding vdWHs, as shown in Figure 4a,b. Not only can we observe the alterations of CBM and VBM in single-layer WSi_2_N_4_ and the alterations of CBM and VBM when vdWHs are formed, but we also discover that the contact types after the formation of single-layer WSi_2_N_4_ and Janus MoSH heterostructures are both p-type ShC, which values are 0.79 eV and 0.34 eV. Notably, the SBHs of MoSH/WSi_2_N_4_, MoHS/WSi_2_N_4_ vdWHs are smaller, indicating that the WSi_2_N_4_ monolayer can be considered as an effective 2D metal contact with the Janus MoSH monolayer.

**Figure 5:**
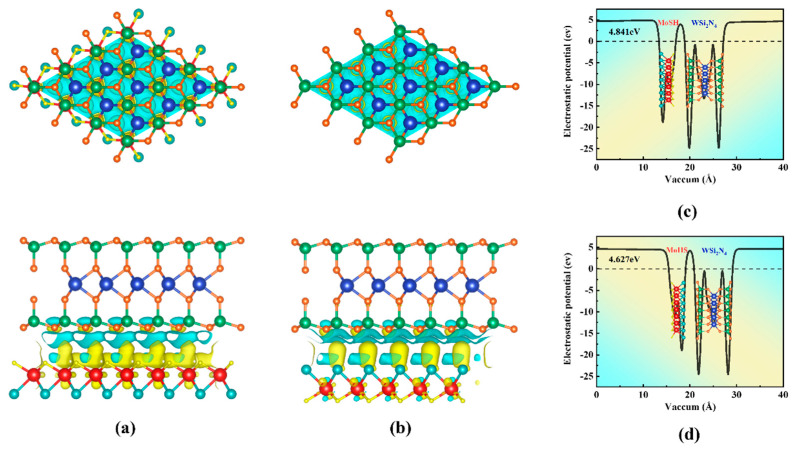
(**a**,**b**) In-plane average charge density difference of Janus MoSH/WSi_2_N_4_, MoHS/WSi_2_N_4_ vdWHs, respectively. (**c**,**d**) In-plane average electrostatic potential of Janus MoSH/WSi_2_N_4_, MoHS/WSi_2_N_4_ vdWHs. The yellow and cyan regions represent charge accumulation and depletion, respectively. The blue, green, orange, red, cyan, and yellow balls represent tungsten, silicon, nitrogen, molybdenum, sulfur, and hydrogen atoms, respectively.

Figure 5a,b display the charge density difference in Janus MoSH/WSi_2_N_4_ vdWHs. The difference in charge density is calculated in the following manner to better understand the charge distribution in Janus MoSH/WSi_2_N_4_ vdWHs [59,60]: Δρ = ρ_vdWHs_ − ρ_MoSH (MoHS)_ − ρWSi_2_N_4_, where ρ_vdWHs_, ρ_MoSH (MoHS)_ and ρWSi_2_N_4_ represent the Janus MoSH/WSi_2_N_4_ vdWHs charge density, isolated Janus MoSH and WSi_2_N_4_ monolayers, respectively. Among them, electron accumulation is represented by the yellow area, while electron depletion is represented by the cyan area. The electron transfer occurring at the contact surface is clearly shown in Figure 5a,b. In short, electrons are consumed in the Si-N layer and accumulated in the Mo-H(Mo-S) layer, and the charge distribution is mostly centered on the contact interface between Janus MoSH and WSi_2_N_4_. As a result, the results imply that the Janus MoSH and WSi_2_N_4_ layers in the corresponding vdWHs exhibit weak interlayer interactions. At the same time, Figure 5c,d illustrate the mean in-plane average electrostatic potential of Janus MoSH/WSi_2_N_4_ vdWHs. It can be observed from Figure 5c,d that after forming the heterostructure, the electrostatic potential of Janus MoSH and MoHS is lower than that of isolated MoSH and MoHS, indicating that electrons are accumulated on the Janus MoSH (MoHS) side and consumed on the WSi_2_N_4_ side. It is transferred from WSi_2_N_4_ to Janus MoSH, and the direction of charge transfer is consistent with Figure 5a,b. In summary, the presence of a built-in electric field is a consequence of interfacial charge transfer. Consequently, the mobility of carriers and the injection of charges may be influenced.

Furthermore, examining the carrier mobility of vdWHs is essential to proving that Janus MoSH/WSi_2_N_4_ vdWHs exhibit favorable characteristics for the development of high-performance optoelectronic devices. It is well known that carrier mobility is a crucial factor to evaluate the conductive properties of optoelectronic materials. The carrier mobility (µ) is inversely proportional to the effective mass (m*) of the carriers, as described by the equation µ = eτ/m*, where e is the electronic charge and τ is the scattering time. This indicates that a lower effective mass leads to higher carrier mobility, given a constant scattering time. Hence, the effective mass plays a critical role in determining the mobility of carriers in the material. Hence, we determine the effective masses of electrons (m_e_*) and holes (m_h_*) by fitting the band-edge dispersion of the VBM and CBM:

$$\frac{1}{m^{*}}=\frac{1}{\hbar^{2}}\times \frac{\partial^{2}E\left(k\right)}{\partial k^{2}}$$

Here, ℏ is the reduced Planck’s constant derived from the Planck constant h (ℏ = h/2π) and k is the wave vector. Our calculated m_e_* and m_h_* of Janus MoSH/WSi_2_N_4_ vdWHs are listed in Table 1. Compared with the traditional semiconductor silicon (m_e_* = 0.81–1.18) [61], it can be found that for Janus MoSH/WSi_2_N_4_ vdWHs (me* = 1.21), the effective mass values of electrons are very close to those of Si, which can prove that Janus MoSH/WSi_2_N_4_ vdWHs have higher carrier mobility. Hence, they have broad application prospects, making them strong contenders for high-speed nano-optoelectronic device applications.

### 2.3. Heterostructures under Interlayer Distance

**Figure 6:**
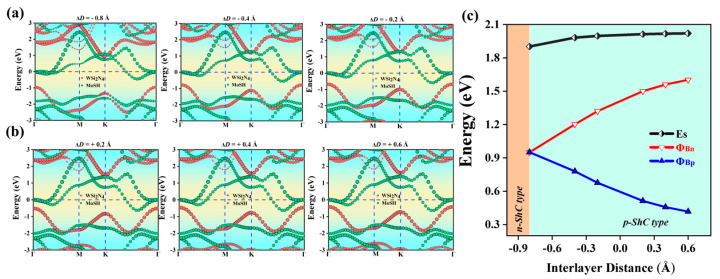
(**a**,**b**) The projected band structure of Janus MoSH/WSi_2_N_4_ vdWHs at different interlayer distances. WSi_2_N_4_ and MoSH monolayers in (**a**,**b**) are separated by red and green circles. (**c**) Evolution of the contact barrier in the Janus MoSH/WSi_2_N_4_ heterostructure with different interlayer distances.

Changing interlayer coupling by applying mechanical strain is a widely recognized method for tuning the interfacial properties of heterostructures. One of the advantages in enhancing the performance of nano-optoelectronic devices is in the tunable SBH and contact type exhibited by Janus MoSH/WSi_2_N_4_ van der Waals heterostructures. Therefore, we have demonstrated the effect of strain engineering through adjustments in the layer distance and the application of an external electric field. It is worth mentioning that the layer distance in 2D-based vdWHs can be tuned by scanning tunneling microscopy [62] or vacuum thermal annealing [63]. Furthermore, we calculated three stacking modes (AA stacking, AB stacking, AC stacking) of Janus MoSH/WSi_2_N_4_ vdWHs under different interlayer distances in Supporting Information Tables S1 and S2, and the findings indicate that MoSH/WSi_2_N_4_ vdWH AA stacking and MoHS/WSi_2_N_4_ vdWH AC stacking have the lowest binding energy. Here, strain is applied by adjusting the layer distance, defined as ΔD = D − D_0_, where the original D values are 2.7 Å and 3.1 Å and D_0_ is the strained interlayer distance. Tensile strain is characterized by the expansion of the layer distance D, whereas compressive strain is characterized by the contraction of D. ΔD < 0 indicates compressive strain, while ΔD > 0 indicates tensile strain. Figure 6a,b show the projected band structures of Janus MoSH/WSi_2_N_4_ vdWHs at different layer distances. We found that with the increase in tensile strain, Φ_Bn_ gradually increased and Φ_Bp_ gradually decreased in a linear relationship. When ΔD > 0, the CBM of the WSi_2_N_4_ layer moves upwards away from the E_F_, leading to an increase in Φ_Bn_. At the same time, the VBM also moves up close to the E_F_, leading to a decrease in Φ_Bp_. The variation in SBH of Janus MoSH/WSi_2_N_4_ vdWHs with ΔD is shown in Figure 6c. When a tensile strain of 0 < ΔD < 0.6 Å is applied, Φ_Bn_ > Φ_Bp_ can be found. In this case, MoSH/WSi_2_N_4_ is of the p-ShC type. However, when a compressive strain of −0.8 < ΔD < 0 Å is applied, it is evident that Φ_Bn_ > Φ_Bp_, still maintaining the p-ShC type. Notably, when a compressive strain of ΔD ≤ −0.8 Å is applied, it is found that Φ_Bp_ eventually becomes bigger than Φ_Bn_; it causes a change from the p-ShC type to the n-ShC type. As a result, by adjusting the layer distance, the SBH and contact type in Janus MoSH/WSi_2_N_4_ vdWHs can be adjusted.

**Figure 7:**
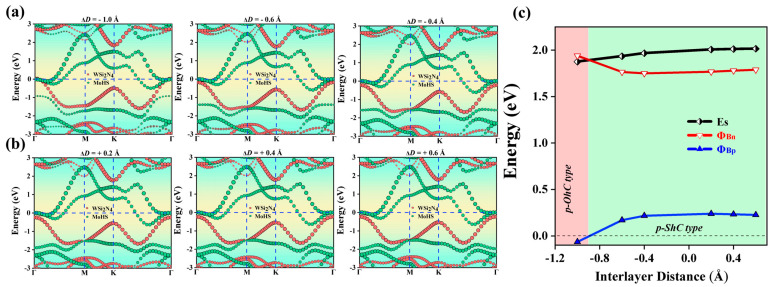
(**a**,**b**) The projected band structures of Janus MoHS/WSi_2_N_4_ vdWHs at different layer distances. The WSi_2_N_4_ and MoHS monolayers in (**a**,**b**) are separated by red and green circles. (**c**) Evolution of the contact barrier in the Janus MoHS/WSi_2_N_4_ heterostructure with different layer distances.

Figure 7a,b show the projected band structure of Janus MoHS/WSi_2_N_4_ vdWHs at different layer distances. We found that the Φ_Bn_ and Φ_Bp_ curves gradually tended to equilibrium with the increase in tensile strain. The variation in SBH of Janus MoHS/WSi_2_N_4_ vdWHs with ΔD is shown in Figure 7c. MoHS/WSi_2_N_4_ has p-ShC type when ΔD > 0 tensile strain is applied. Furthermore, when a compressive strain of −0.9 < ΔD < 0 Å is applied, the CBM of the WSi_2_N_4_ layer can be found to shift upwards away from the E_F_, leading to an increase in Φ_Bn_. At the same time, the VBM also moves up close to the E_F_, leading to a decrease in Φ_Bp_. Since Φ_Bn_ > Φ_Bp_, the p-ShC type is still maintained. However, when a compressive strain of ΔD ≤ −0.9Å is applied, it can be found that the VBM of the WSi_2_N_4_ layer moves upwards across the E_F_, forming a transition from p-ShC type to p-OhC type. Therefore, the SBH in Janus MoHS/WSi_2_N_4_ vdWHs can be tuned by changing the layer distances, but the contact type does not change.

### 2.4. Heterostructures under Electric Field

**Figure 8:**
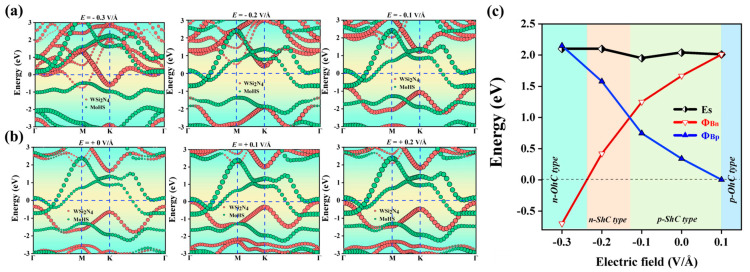
Projected band structures of Janus MoSH/WSi_2_N_4_ vdWHs along the z direction under different applied electric fields in (**a**,**b**). WSi_2_N_4_ and MoSH monolayers are separated by red and green circles, respectively. (**c**) Evolution of the contact barrier in Janus MoSH/WSi_2_N_4_ vdWHs under different electric fields.

**Figure 9:**
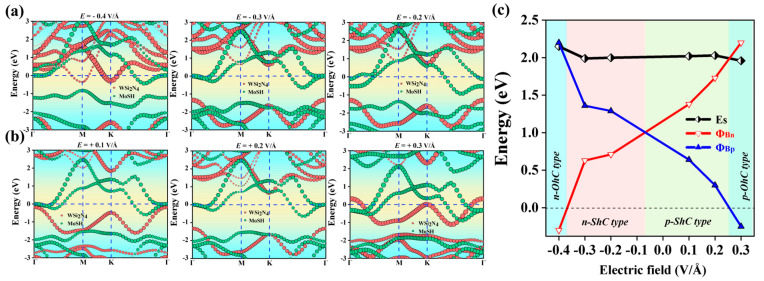
Projected band structures of Janus MoHS/WSi_2_N_4_ vdWHs along the z direction under different applied electric fields in (**a**,**b**). WSi_2_N_4_ and MoHS monolayers are separated by red and green circles, respectively. (**c**) Evolution of the contact barrier in Janus MoHS/WSi_2_N_4_ vdWHs under different electric fields.

The investigation focused on analyzing the impact of the electric field on the electronic characteristics and contact types of Janus MoSH/WSi_2_N_4_ van der Waals heterostructures (vdWHs), as depicted in Figure 8 and Figure 9. We can observe that the SBH of Janus MoSH/WSi_2_N_4_ vdWHs varies linearly with an electric field. Here, an external electric field is imposed on vdWHs in the z direction. Figure 8a,b show that for Janus MoSH/WSi_2_N_4_ vdWHs, when a positive electric field is introduced, the conduction band minimum of the WSi_2_N_4_ layer is displaced in an upward direction, away from the E_F_, resulting in an elevation of the barrier height Φ_Bn_. In contrast, the valence band maximum exhibits an upward shift in proximity to the E_F_, leading to a reduction in Φ_Bp_. Furthermore, when a negative electric field is imposed, the conduction band minimum of the WSi_2_N_4_ layer undergoes a downward displacement, approaching the E_F_. Consequently, this results in a decrease in Φ_Bn_. In contrast, the valence band maximum exhibits a downward shift relative to the E_F_, leading to an increase in Φ_Bp_. Figure 8c exhibits notable variations in SBH and contact type. When a negative electric field around −0.37 < E < −0.07 V/Å is imposed, Φ_Bp_ > Φ_Bn_ can be found. In this case, the n-ShC type exists in Janus MoSH/WSi_2_N_4_ vdWHs. When the magnitude of the electric field becomes around −0.37 V/Å, the CBM of the WSi_2_N_4_ layer shifts down across the E_F_ and Φ_Bn_ decreases to 0, which indicates that Janus MoSH/WSi_2_N_4_ vdWH form changes from n-ShC type to n-OhC type. Moreover, the application of an electric field within the range −0.07 < E < 0.26 V/Å is imposed; Φ_Bn_ increases while Φ_Bp_ declines, and Φ_Bn_ progressively grows to be larger than Φ_Bp_. Consequently, it was shown that Janus MoSH/WSi_2_N_4_ vdWH form changes from n-ShC type to p-ShC type. When the magnitude of the electric field becomes around 0.26 V/Å, the VBM of the WSi_2_N_4_ layer moves upward across the E_F_, and the observed phenomenon is that Φ_Bp_ decreases to 0; this suggests that there is a transition in the Janus MoSH/WSi_2_N_4_ vdWHs from a p-ShC type to a p-OhC type. Likewise, the projected band structure of Janus MoSH/WSi_2_N_4_ vdWHs along the z direction under varying applied electric fields is shown in Figure 9a,b. For Janus MoSH/WSi_2_N_4_ vdWHs, when a positive electric field is introduced, the conduction band minimum of the WSi_2_N_4_ layer is displaced in an upward direction, away from the E_F_, resulting in an elevation of the barrier height Φ_Bn_. In contrast, the valence band maximum exhibits an upward shift in proximity to the E_F_, leading to a reduction in Φ_Bp_. Furthermore, when a negative electric field is imposed, the conduction band minimum of the WSi_2_N_4_ layer undergoes a downward displacement, approaching the E_F_. Consequently, this results in a decrease in Φ_Bn_. In contrast, the valence band maximum exhibits a downward shift relative to the E_F_, leading to an increase in Φ_Bp_. When a negative electric field around −0.24 < E < −0.14 V/Å is imposed, Φ_Bp_ > Φ_Bn_ can be found. In this instance, the n-ShC type exists in Janus MoHS/WSi_2_N_4_ vdWHs. Notably, when the magnitude of the electric field becomes around −0.24 V/Å, the CBM of the WSi_2_N_4_ layer shifts down across the E_F_ and Φ_Bn_ decreases to 0, which indicates that the Janus MoHS/WSi_2_N_4_ vdWH form changes from n-ShC type to n-OhC type. Moreover, the application of an electric field within the range −0.14 < E < 0.1 V/Å is imposed; Φ_Bn_ increases while Φ_Bp_ decreases, and Φ_Bn_ progressively grows to be larger than Φ_Bp_. Consequently, it was shown that Janus MoHS/WSi_2_N_4_ vdWH form changes from n-ShC type to p-ShC type. When the magnitude of the electric field becomes around 0.1 V/Å, the VBM of the WSi_2_N_4_ layer moves upward through the E_F_ and the observed phenomenon is that Φ_Bp_ decreases to 0; this suggests that there is a transition in the Janus MoHS/WSi_2_N_4_ vdWH form from a p-ShC type to a p-OhC type. The aforementioned findings demonstrate that the contact type and SBH of Janus MoSH/WSi_2_N_4_ and MoHS/WSi_2_N_4_ vdWHs may be modulated through the application of an electric field. Additionally, these heterostructures exhibit a transition from n-type Schottky contact to p-type Schottky contact, as well as a transformation from Schottky contact to Ohmic contact. The results of our study have the potential to offer novel opportunities for the future development of high-performance nanodevices utilizing metal/semiconductor Janus MoSH/WSi_2_N_4_ vdWHs.

## 3. Computational Methods

The density functional theory framework is utilized for conducting geometry structural optimization and electronic performance calculations [64]. These calculations are carried out using the Vienna ab initio simulation package (VASP) [65], which incorporates the projector-augmented plane wave (PAW) approach [66] to account for the ion–electron interaction. Visualization for Electronic Structural Analysis (VESTA ver. 3.5.8) [67] is a software tool that is utilized for processing various types of data related to structural models, volumetric information such as electron and nuclear densities, and crystal morphologies. The electronic exchange correlation functional is treated using the generalized gradient approximation (GGA) [68] in the form proposed by Perdew, Burke, and Ernzerhof (PBE) [69]. The energy cutoff of the plane waves is set to 550 eV, with an energy precision of 10^−6^ eV. The atomic locations undergo complete relaxation until the magnitude of the force acting on each atom is below 10^−3^ eV/Å. A Monkhorst-Pack k-point grid with a KSPACING value of 0.15 Å^−1^ is employed in the calculations. The supercell approach is commonly used to model monolayers, where a vacuum separation of about 40 Å is implemented to mitigate the effects of interactions between adjacent layers. Since the generalized gradient approximation (GGA) usually underestimates the bandgap, we chose to use the Heyd−Scuseria−Ernzerhof (HSE06) hybrid functional [70] to calculate the band structure. Dynamic stabilities and phonon dispersion curves are computed with the supercell approach, as implemented in the Phonopy code [71]. The dipole correction was also included in the calculations.

## 4. Conclusions

To summarize, we investigated the electronic structure and interfacial properties of the emerging two-dimensional metal/semiconductor Janus MoSH/WSi_2_N_4_ vdWHs through first-principles calculations. Janus MoSH/WSi_2_N_4_ vdWHs retain the metallic properties of a single layer of Janus MoSH and the intrinsic semiconductor properties of a single layer of WSi_2_N_4_. In this study, we provide evidence that both Janus MoSH/WSi_2_N_4_ and MoHS/WSi_2_N_4_ exhibit p-type Schottky contacts of the SBH, measuring 0.79 eV and 0.34 eV, respectively. These findings suggest advantages in enhancing the efficiency of charge injection. Furthermore, we conducted an investigation into the electronic structure and SBH phenomena at varying interlayer distances and electric field strengths, and the tunable SBH can lead to faster response times and higher sensitivity. The ability to tune the electronic properties and interfacial characteristics through layer spacing and electric fields can significantly enhance device performance. The findings indicate that the electronic characteristics and interfacial contact of Janus MoSH/WSi_2_N_4_ vdWHs can be adjusted by changing the layer distance and applying an electric field. These adjustments not only influence the SBH, but also lead to the transformation from p-type Schottky contact to n-type Schottky contact, and the transition from ShC to Ohc. Our findings not only contribute to the fundamental understanding of Janus MoSH/WSi_2_N_4_ vdWHs but also provide a pathway for their integration into high-performance optoelectronic devices.

## Figures and Tables

**Table 1:** Calculated lattice parameters (a), interlayer distance (D), band gap (E_g_) obtained by PBE calculations, and effective mass for electrons (m_e_^x^) and holes (m_h_^y^) along the x and y directions.

	a (Å)	D (Å)	E_g_ (eV)	m_e_^x^/m_0_	µ_e_ (cm^2^/Vs)	m_h_^y^/m_0_	µ_h_ (cm^2^/Vs)	Contact Types
1T-WSi_2_N_4_	2.91	-	2.03	0.36		1.35		-
MoSH/WSi_2_N_4_	2.91	2.74	2.01	1.21	145	1.05	96	p-ShC
MoHS/WSi_2_N_4_	2.91	3.15	2.02	1.84	167	1.17	150	p-ShC

## Data Availability

The data that support the findings of this study are available from the corresponding authors upon reasonable request.

## Supplementary Materials

The following supporting information can be downloaded at: https://www.mdpi.com/article/10.3390/molecules29153554/s1, Figure S1. Results of Orientation-dependent Young’s modulus and Poisson’s ratio for monolayer Janus MoSH and WSi_2_N_4_. Tables S1 and S2. Calculation of binding energy of Janus MoSH/WSi_2_N_4_ heterostructures under modifying the interlayer distance(ΔD) for different stacking modes.
