# Supplementary material for: Janus MoSH/WSi2N4 van der Waals Heterostructure: Two-Dimensional Metal/Semiconductor Contact
Source: Molecules. 2024 Jul 28;29(15):3554. doi: 10.3390/molecules29153554 (PMC11313900; doi:10.3390/molecules29153554)
Supplement: Supplementary file 1 [file molecules-29-03554-s001.zip › molecules-3066593-supplementary.pdf]

Supplementary Online Material

Article

Janus MoSH/WSi_2N_4 van der Waals Heterostructure: Two-Dimensional Metal/Semiconductor Contact

Yongdan Wang [1,2,†], Xiangjiu Zhu [1,3,†], Hengshuo Zhang [1], Shitong He [1], Ying Liu [1], Wenshi Zhao [1], Huilian Liu [1,*] and Xin Qu [1,*]

[1] Key Laboratory of Functional Materials Physics and Chemistry of the Ministry of Education, Jilin Normal University, Changchun 130103, China; ydwang@jlnu.edu.cn (Y.W.); zxj1474131433@126.com (X.Z.); jlnuzhs@163.com (H.Z.); m15333854769@163.com (S.H.); 233120967@mails.jlnu.edu.cn (Y.L.); 2405006@jlnu.edu.cn (W.Z.)
[2] School of Foreign Languages, Jilin Normal University, Siping 136000, China
[3] State Key Laboratory of Superhard Materials, College of Physics, Jilin University, Changchun 130012, China
* Correspondence: lhl541@jlnu.edu.cn (H.L.); quxin515@163.com (X.Q.)
† These authors contributed equally to this work.

Keywords: two-dimensional heterostructures; first-principles calculations; electronic properties; electrical contact

1. Orientation-dependent Young's modulus and Poisson's ratio 2
2. Calculate the binding energy for different stacking modes and interlayer distances 3

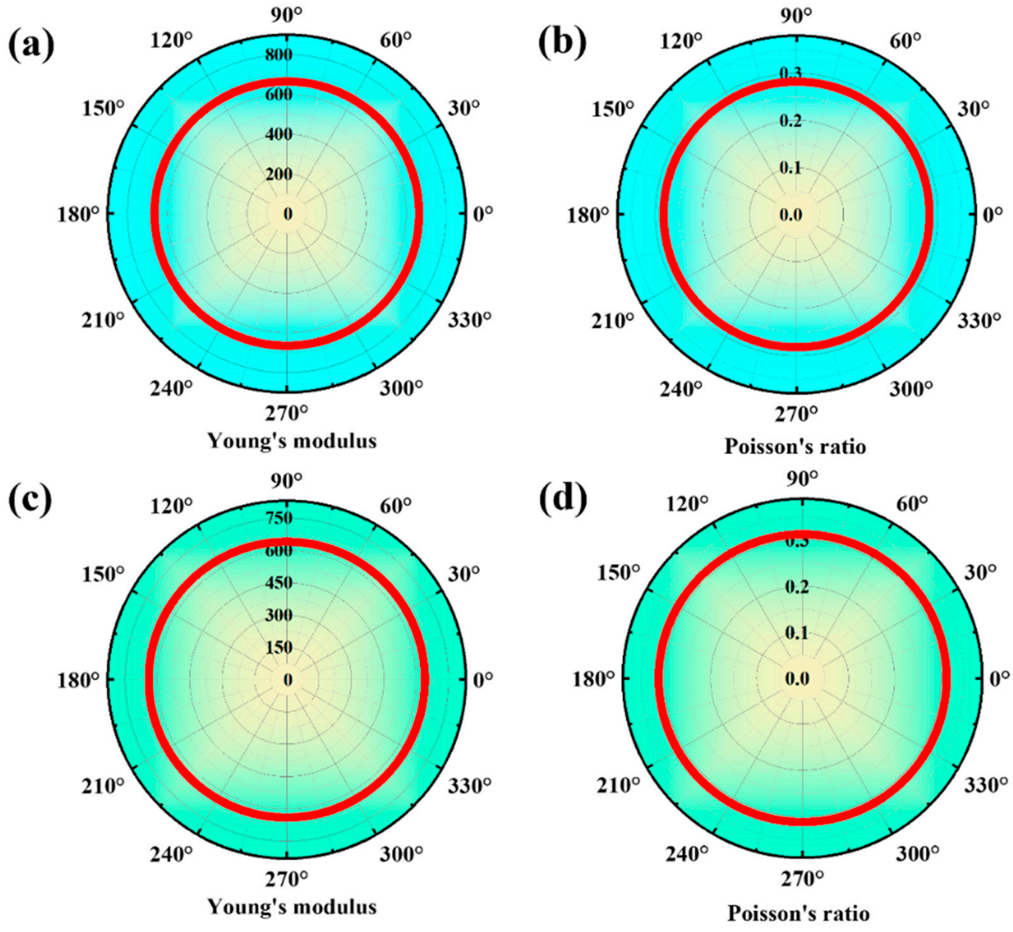

Figure S1. Polar plots of (a) Young's modulus and (b) Poisson's ratio for ground state Janus MoSH/WSi_2N_4 vdWHs. Polar plots of (c) Young's modulus and (d) Poisson's ratio of Janus MoHS/WSi_2N_4 vdWHs in the ground state.

Table S1. Calculation of binding energy of Janus $MoSH/WSi_2N_4$ heterostructures under modifying the interlayer distance(ΔD) for different stacking modes.

ΔD (Å)	Binding Energy (eV)		
stacking modes	AA	AB	AC
0	**-8.43073**	-8.43072	-8.28666
0.2	-8.39863	-8.39838	-8.28293
0.4	-8.39715	-8.39705	-8.28043
0.6	-8.39541	-8.39538	-8.27809
0.8	-8.39362	-8.39363	-8.27595
1.0	-8.39192	-8.39194	-8.27403

Table S2. Calculation of binding energy of Janus $MoHS/WSi_2N_4$ heterostructures under modifying the interlayer distance(ΔD) for different stacking modes.

ΔD (Å)	Binding Energy (eV)		
stacking modes	AA	AB	AC
-0.2	-8.39931	-8.39391	-8.40045
0	-8.43147	-8.39697	**-8.43225**
0.2	-8.39875	-8.43148	-8.39942
0.4	-8.39727	-8.39656	-8.39771
0.6	-8.39632	-8.39518	-8.39581
0.8	-8.39556	-8.39361	-8.39393
1.0	-8.39380	-8.39212	-8.39216
